# Offshore Wind Energy Climate Projection Using UPSCALE Climate Data under the RCP8.5 Emission Scenario

**DOI:** 10.1371/journal.pone.0165423

**Published:** 2016-10-27

**Authors:** Markus Gross, Vanesa Magar

**Affiliations:** Departamento de Oceanografía Física, Centro de Investigación Científica y de Educación Superior de Ensenada, Ensenada, BC, Mexico; Universidade de Vigo, SPAIN

## Abstract

In previous work, the authors demonstrated how data from climate simulations can be utilized to estimate regional wind power densities. In particular, it was shown that the quality of wind power densities, estimated from the UPSCALE global dataset in offshore regions of Mexico, compared well with regional high resolution studies. Additionally, a link between surface temperature and moist air density in the estimates was presented. UPSCALE is an acronym for *U*K on *P*RACE (the Partnership for Advanced Computing in Europe)—weather-resolving *S*imulations of *C*limate for glob*AL* *E*nvironmental risk. The UPSCALE experiment was performed in 2012 by NCAS (National Centre for Atmospheric Science)-Climate, at the University of Reading and the UK Met Office Hadley Centre. The study included a 25.6-year, five-member ensemble simulation of the HadGEM3 global atmosphere, at 25km resolution for present climate conditions. The initial conditions for the ensemble runs were taken from consecutive days of a test configuration. In the present paper, the emphasis is placed on the single climate run for a potential future climate scenario in the UPSCALE experiment dataset, using the Representation Concentrations Pathways (RCP) 8.5 climate change scenario. Firstly, some tests were performed to ensure that the results using only one instantiation of the current climate dataset are as robust as possible within the constraints of the available data. In order to achieve this, an artificial time series over a longer sampling period was created. Then, it was shown that these longer time series provided almost the same results than the short ones, thus leading to the argument that the short time series is sufficient to capture the climate. Finally, with the confidence that one instantiation is sufficient, the future climate dataset was analysed to provide, for the first time, a projection of future changes in wind power resources using the UPSCALE dataset. It is hoped that this, in turn, will provide some guidance for wind power developers and policy makers to prepare and adapt for climate change impacts on wind energy production. Although offshore locations around Mexico were used as a case study, the dataset is global and hence the methodology presented can be readily applied at any desired location.

## Introduction

The special report by the Intergovernmental Panel on Climate Change (IPCC) Working Group III, on Renewable Energy Sources and Climate Change Mitigation (SRREN) [1], states that “research to date suggests that climate change is not expected to greatly impact the global technical potential for wind energy development but changes in the regional distribution of the wind energy resource may be expected”. This conclusion seems well supported by previous work [2], where the authors provide substantial background information on the use of climate models to estimate the impact of climate change on wind energy resources. Whilst mainly focusing on Europe, in that work it is argued that moderate reductions on resources are expected, with a reduction of approximately 3% over the next 50 years, and a reduction of approximately 5% over the next 100 years. In contrast, in another study for the USA [3], utilizing a 2.5° Hadley Centre Coupled Model (HadCM) II, a reduction of 10 to 15% in mean wind speeds was predicted, corresponding to a 30 to 40% wind energy resource reduction. However, it is difficult to make comparisons between these studies, due to significant differences on how they were performed and differences in the climate model outputs.

Without spatial downscaling, the resolution of these global models would not be sufficient for wind energy applications, and therefore a downscaling method needs to be adopted. In a previous study [4], statistical downscaling methods were used to increase the resolution of the global climate models created for the IPCC (with highest resolution of 1.9°), and assess the impact of climate change on wind speeds in the North West of the USA. Their results suggest a seasonal component of the climate change impact (under the Special Report on Emissions Scenarios A1B and A2 [5]), with summertime wind speeds decreasing by 5-10%, and low or no impact on winter months. As a consequence, a 40% reduction in summertime generation potential is projected at typical turbine hub heights. This work clearly shows that higher resolution models have to be used in order to provide suitable, less ambiguous results. This is because coarse resolution models do not capture well the spatial inhomogeneity of the wind energy potential.

It is important to note here that over the years there has been a continuous improvement in resolution of both regional (RCMs) and global (GCMs) climate models, mainly due to the increase of available computer resources and advances in numerical schemes to solve the underlying equations. Hence, what used to be the resolution of a RCM is now the resolution of a GCM. Despite such improvements, some authors [6] state that the resolution of atmosphere-ocean global climate models is still inappropriate to accurately characterize wind climates, and suggest to utilize a RCM model with a resolution of 0.44° × 0.44°, primarily in the areas of interest. For example, Pryor, Barthelmie and Kjellström [7] present results for Europe, analyzing the climate change impact of the A2 scenario using a RCM with a (highest) resolution of 0.44° × 0.44°. It is emphasized that much of the solution in the RCM in these studies is dominated by the lateral boundary conditions (LBC), which leads to inaccuracies in the model predictions because the LBC derived from a low resolution GCM lacks the small scales, and the RCM will have to generate them; this requires larger computer capabilities to handle the additional domain and time scales. Therefore, if it is available, a high resolution GCM model, such as that for the UPSCALE project [8, 9], would be preferable.

Pryor, Schoof and Barthelmie [10] perform an empirical downscaling on GCM results for Northern Europe, with the finest resolution of 1.875° × 1.875°. Again, as for previous examples using models with such a coarse resolution, downscaling is required. The authors analysed the A2 emission scenario, and significant changes in wind energy production are reported. The A2 scenario equates to a moderate to high greenhouse gas cumulative emissions, resulting in global carbon dioxide emissions in 2100 that are almost four times the 1900 value [5]. As reported in that work, the downscaled mean and the 90^*th*^ percentile wind speed over Northern Europe during the 21^*st*^ century are likely to differ from those that prevailed during the end of the 20^*th*^ century by less than ±15%. This change in the wind speed signal is currently comparable to variations in downscaling results between GCM models, due to variations in methods and setups used in the downscaling exercises. Therefore, the level of uncertainty associated with downscaling procedures is high, and predictions of downscaled models that do quantify the uncertainty using, for example, ensemble realizations, need to be interpreted with caution.

At coarse resolution the general circulation is well resolved, explaining the success of early climate models. However, closer to the surface, the winds are strongly influenced by the presence of land masses and by differences in sea surface temperature. Hence, the need for increased resolution is intrinsic to the wind energy problem: while the general circulation of the atmosphere is fairly insensitive to small changes in wind speeds, the wind power density will change significantly if the wind speeds change by a few meters per second. Furthermore, for the results to be relevant to actual wind energy projects the locations identified as suitable for exploitation have to be within physical reach, that is, sufficiently close to cities and energy distribution infrastructure. In low resolution studies, the geographic margin of error is large, in many cases too large for wind energy applications, as the potential predicted by the model may not be achieved in the commercially viable locations. Indeed, differences in several hundred kilometers may not be large in terms of global general circulation of the atmosphere, but they do make a significant difference when analyzing the economic viability of wind energy projects through numerical wind energy resource assessments.

In this paper the impact of climate change on offshore wind energy resources around Mexico under the RCP8.5 scenario [11–13] is presented. It is worth noting that using Mexico as a case study is by no means a limitation, as the UPSCALE dataset is global and the methodology is not region-specific. Here the land area was excluded from the results presented as it is expected that local orography will have a strong effect in the area of interest for wind energy, corresponding to the first couple of hundred meters above land level. Indeed, orography is not resolved well enough even with this high resolution dataset. Therefore, in order to prevent the distribution of unreliable and potentially misleading data, the land area is not analysed here.

## The UPSCALE climate dataset

The dataset (available from http://services.ceda.ac.uk/cedasite/resreg/application?attributeid=gws_upscale), described and documented in detail in previous publications [8, 14], is based on the HadGEM3 Global Atmosphere 3 (GA3) and Global Land 3 (GL3) configurations of the MetUM and the Joint UK Land Environment Simulator (JULES). The UPSCALE simulations use a 25km to 32km grid, which is around 1/3°, with 85 vertical levels, with the uppermost level at 85km. Velocity data is recorded at three-hourly intervals.

The future climate simulations were configured with sea surface temperature (SST) from the Operational Sea Surface Temperature and Sea Ice Analysis (OSTIA) dataset [9]. The OSTIA dataset used in the present climate runs has a native resolution of 1/20°, and takes into account the SST change between 2000 and 2100 under the RCP8.5 climate change scenario, obtained with the Hadley Centre Global Environmental Model version 2 Earth System (HadGEM2-ES) model [12, 13]. The SST change was calculated for each month, then interpolated in space and time, and added to the daily variations of the OSTIA forcing data. The initial conditions for the two ensemble runs performed here (one five-member set for the present climate and one three-member set for the future climate) were taken from consecutive days of a test configuration [8], and were run for 25.6 years.

## Methodology

The key quantity computed in the previous study, [15], is the Wind Power Density (WPD). If the temporal sampling is sufficient, then the WPD can be computed as

$$\begin{array}{c} WPD=\frac{1}{2n}\sum_{t=1}^{n}\rho_{t}u_{t}^{3}, \end{array}$$ (1)

where *n*, *ρ* and *u* denote the number of (time) samples, air density at time *t* and speed at time *t*, respectively. Here *n* = 74880 samples were considered, all taken at three-hourly intervals. The temporal sampling length is adequate if the value of *WPD* is independent of the length of the time series. Below it is shown that doubling the size of the time series does not change the results greatly, and therefore the sampling length is deemed sufficient. Then, the relative difference between ensemble (ens) and reference (ref) datasets was computed according to

$$\begin{array}{c} \delta_{rel}=\frac{WPD_{ens}-WPD_{ref}}{WPD_{ref}}, \end{array}$$ (2)

for each of the ensemble members *WPD*_*ens*_. The reference Wind Power Density (*WPD*_*ref*_) is that presented by Gross and Magar in [15]. It corresponds to the unperturbed climate between February 1985 and December 2011. From the relative difference (Eq 2), the root mean square (RMS) error, *RMS*_*rel*_, is computed as

$$\begin{array}{c} RMS_{rel}=\sqrt{\frac{1}{n}\sum \delta_{rel}^{2}}. \end{array}$$ (3)

As stated above, the first question to be answered is whether the time series is sufficiently long. This is achieved by first showing that an extended dataset obtained by combining two ensemble members can be treated as an independent measurement. Combining here means that a new, longer time series is created by concatenating two ensemble members. Independent from the other ensemble members means that, whilst sampling the same climate of course, the new time series can be seen as sampling the weather before or after the time series used for comparison. The order of samples in the new time series does not impact on the result. Four ensemble members *f*, *g*, *h*, and *i*, are used to answer this question. From these four members, only three unique combinations can be formed: *h* + *g*, *i* + *g* and *i* + *h*. The ensemble member *f* is used to extend the reference dataset. Once this is established, the result of using the new (or extended) time series is compared to the result using a single time series. It is expected that the extension of the time series will make a difference, but this difference should be small. If a time series is sufficiently long to *fully* capture the climate, then an extended time series representing the same climate will not generate statistically significant differences between measurements. To test whether measurement differences are statistically significant, the Mann-Whitney-Wilcoxon (MWW) Rank-sum test [16, 17] is used. As the magnitude of the measurement differences was found to be small, the sampling length of the 25.6-year long time series is deemed sufficient for Eq 1 to generate a robust result.

For the second question being addressed here, regarding the impacts of climate change on wind energy resources, the future climate dataset is compared and contrasted to the present climate dataset. It is worth recalling that in the UPSCALE dataset there is only one future climate change scenario available, corresponding to the RCP8.5 scenario. For the comparison between the two datasets to be meaningful, the following criteria have to be fulfilled:

- The difference between the climate change signal and any of the present climate signals, has to be greater than the difference between any of the present climate signals by the ensemble members *f*, *g*, *h* and *i* analysed previously.
- The measure for comparison is the RMS relative difference to each of the ensemble members.
- When this can be assumed to be normally distributed a 2*σ* difference is considered significant.

To show this, the first step is to establish whether the obtained RMS values can be assumed to follow a normal distribution. This is achieved using the Shapiro-Wilks test. Then the difference between the RMS values for the different time series (original, present climate ensemble, extended and future climate signals) can be compared to the standard deviation of the data. A difference of more than 2*σ* in the RMS values would indicate a statistically significant difference between datasets.

## Results

**Fig 1:**
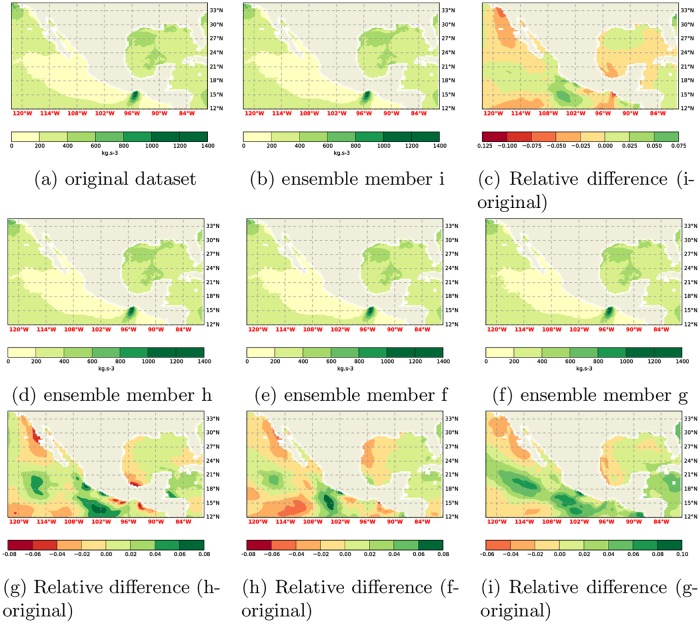
Comparison of WPD at 50m. Computations using the present climate ensemble members and the original dataset from Gross and Magar, [15].

Field plots for a hub height of 50m are shown in Fig 1.

The respective plots for 10m and 150m hub heights are omitted because they differ only slightly.

**Fig 2:**
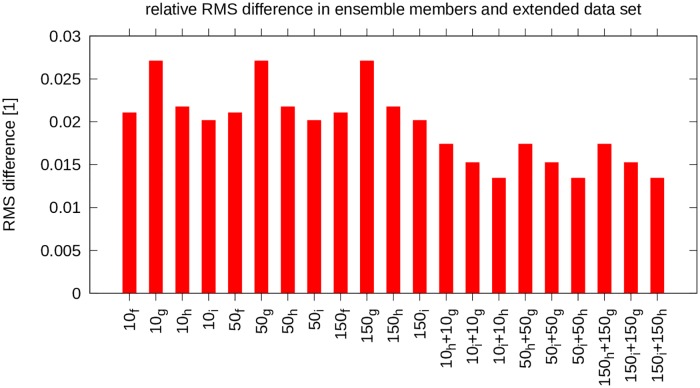
Relative RMS difference (cf. Eq 4) for ensemble members and extended dataset. Only grid points in the study area contributed to the RMS. Each grid point was treated equally due to the small variation of grid box size over the study area. The non-extended datasets are compared to the original dataset, and the extended datasets are compared to the original dataset extended by ensemble member *f*.

The results for the relative RMS difference are shown in Fig 2. The RMS differences are labeled according to the hub height and the ensemble member, i.e. 10_*f*_ represents the difference for the 10m hub height, ensemble member *f*.

### Extended datasets

An ensemble member can be seen as an independent set of measurements. This means that also any combination of ensemble members is a new set of measurements. Using these extended sets, generated by combining ensemble members, the effect of a dataset of twice the length can be investigated. Using the original dataset augmented with ensemble member *f* as control (*WPD*_*ref*_), three unique datasets can be computed using the *g* + *h*, *g* + *i* and *h* + *i* combinations. The RMS difference,

$$\begin{array}{c} RMS_{rel,xy}=\sqrt{\frac{1}{n}\sum {\left.(\frac{WPD_{x+y}-WPD_{ref}}{WPD_{ref}}\right)}^{2}} \end{array},$$ (4)

with *x* and *y* being placeholders for the ensemble members combined when constructing the time series, for these three sets (*RMS*_*rel*, *hg*_, *RMS*_*rel*, *ig*_ and *RMS*_*rel*, *ih*_) is (slightly) lower than the *RMS*_*rel*_ for the original (non-extended) sets; see Fig 2 for a comparison. This is as expected, as more data is being used.

Then, the Mann-Whitney-Wilcoxon (MWW) rank-sum test [16, 17] applied to both the original and the extended datasets yields an identical *p*_*MWW*_ value of 0.03389 for the 10m, 50m and 150m results. Therefore, since *p*_*MWW*_ ≤ 0.05, it can be claimed that the distributions differ significantly (even though *p*_*MWW*_ is not much smaller than 0.05), and therefore that extending the dataset has a statistically significant impact on the difference. Comparing the extended datasets, 0.0174 > *RMS*_*rel*_ > 0.0134, with the original dataset, 0.027 > *RMS*_*rel*_ > 0.0201 (the first 12 plots, 10_*f*_ to 150_*i*_, in Fig 2), shows that the error is of the same order of magnitude as that for the ensemble members. At this level it is likely that other factors in the methodology are more important, such as the extrapolation, the resolution and the parameterizations used in the GCM.

### The future climate dataset

**Fig 3:**
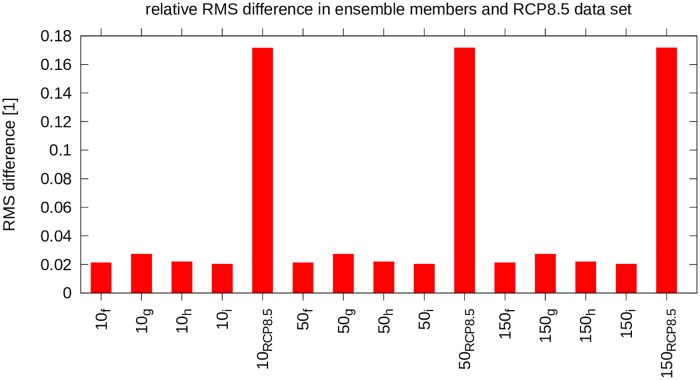
Relative RMS difference for ensemble members and climate change dataset.

**Fig 4:**
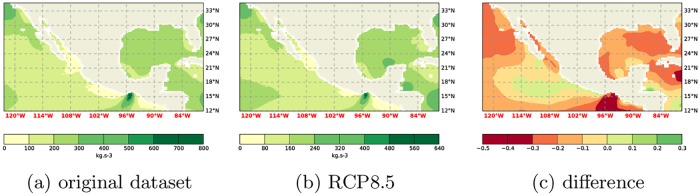
Comparison of WPD computations at 10m using the RCP8.5 and the original dataset. a) Original dataset following Gross and Magar [15], b) RCP8.5 dataset, c) relative difference.

**Fig 5:**
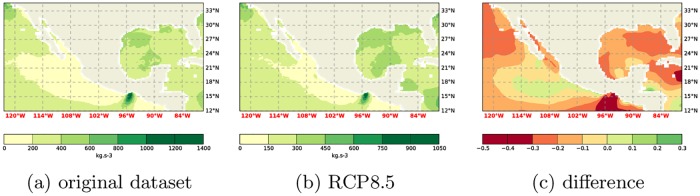
Comparison of WPD computations at 50m using the RCP8.5 and the original dataset. a) Original dataset following Gross and Magar [15], b) RCP8.5 dataset, c) relative difference.

**Fig 6:**
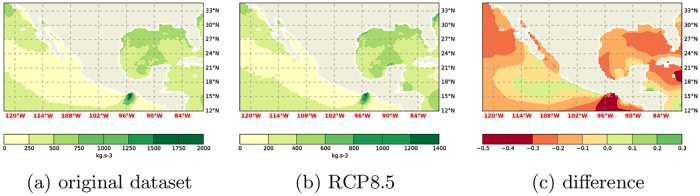
Comparison of WPD computations at 150m using the RCP8.5 and the original dataset. a) Original dataset following Gross and Magar [15], b) RCP8.5 dataset, c) relative difference.

With reasonable confidence the benefit of using more data is small/negligible, and hence that the data from one run already provides good guidance, the focus can now shift to the analysis of the future climate data. It can be seen that the difference between the RCP8.5 climate and the current climate is substantial (Fig 3). Indeed, the difference is larger than expected from the natural variability of the climate system. This natural variability manifests itself, amongst other things, in the RMS difference between the ensemble members. Also, the *W* values of the Shapiro-Wilks test [18] (see Table 1) indicate a normal distribution of the RMS differences of the original dataset and the ensemble members. With a 95% confidence interval the critical *W* value is 0.748. Here the *W* values are greater than the critical *W* and *p*_*SW*_ > *α* = 0.05. Therefore the data is likely normally distributed. Assuming it is normally distributed, a deviation of 2*σ* is statistically relevant. Comparing the future climate run to the present climate run shows a difference of ≈ 25*σ*. It can therefore be safely said that this difference is statistically relevant. The field plots of the difference, the RCP8.5 future climate versus the original climate, is reproduced in Figs 4, 5 and 6.

**Table 1:** Shapiro-Wilks test results and standard deviation of the RMS differences.

height	*W*	*p*_*SW*_	2*σ*
10	0.81538	0.132792	0.005419
50	0.81543	0.132898	0.005418
150	0.81549	0.133035	0.005417

### Regional results

**Fig 7:**
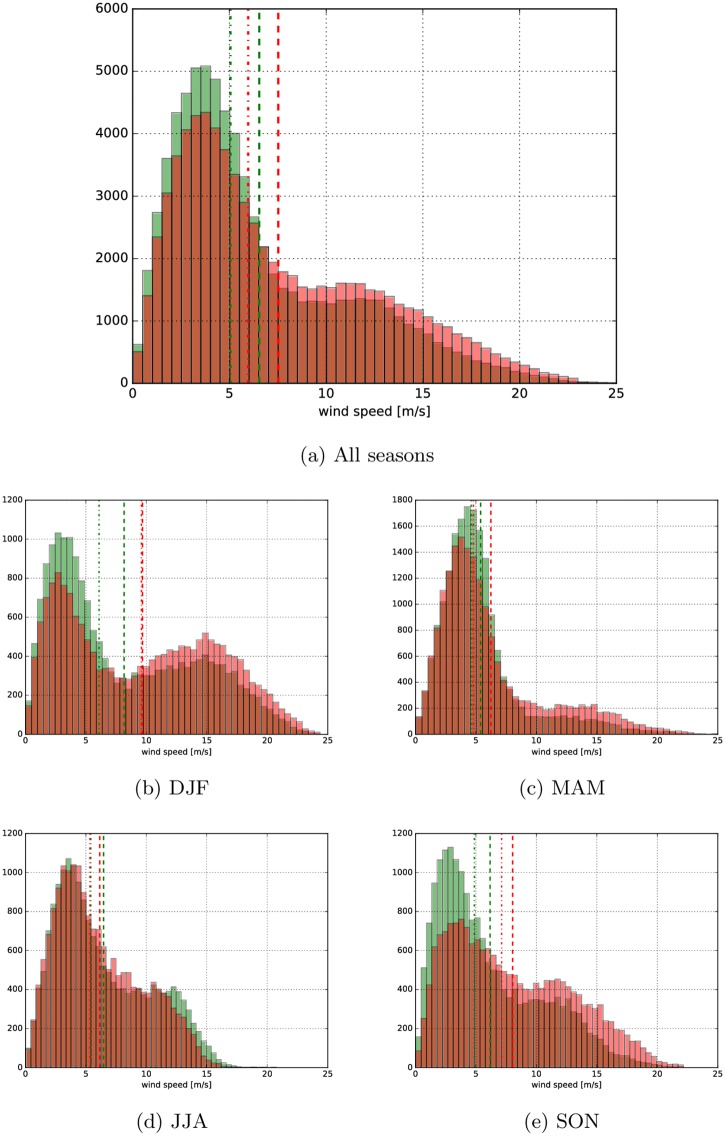
Wind speed histogram for the Gulf of Tehuantepec (latitude = 15.2344°N, longitude = 265.078°W). Present climate in red and RCP8.5 climate in green, mean is indicated with a dashed and median with dash-dotted line. The vertical axis shows event count (here using the three-hourly sampled dataset) over the 25.6-year period.

Whilst the regional results are only of relevance to the developers and policy makers in the respective region, there is still general interest in what information can be obtained from the global dataset. For example, the wind speed histogram for the Gulf of Tehuantepec is shown here in Fig 7, where the future climate signal indicates a reduction of 50% in the WPD. The present climate dataset is shown in red and the RCP8.5 dataset in translucent green. It is interesting to note that there is a *reduction* in high wind events, and an increase in below average wind speed events. This is both, good news to the developers and contrary to the general popular assumption (which is generally both over-interpreted and over-generalized) that with future climate extreme events will occur more frequently.

## Conclusion

Here it was shown that the altered climate of a high resolution GCM simulation does have a marked impact on the projected wind power density for offshore locations around Mexico. As far as the climate results can be relied on, and there obviously is still a lot of discussion and ongoing work in the scientific community, this will provide important insights in the climate change scenarios than may become reality.

It was also shown that using one time series of 74880 samples (≈ 25.6 years with a three-hourly sampling rate) already produces a reasonably solid estimate. Doubling the sample size did improve the results, in a statistically significant way, however, only a modest difference was observed, certainly in contrast to the response to the climate change run.

From an environmental perspective it is disappointing to see the broad scale drop in projected average wind power densities. However, sensible technical and financial decisions should be able to incorporate or mitigate this. In any case, as well as the uncertainties underlying this analysis (small size, resolution, extrapolation, etc.), it is not given that the future climate will exactly instantiate the RCP8.5 climate change scenario. Baring this in mind, it is still worth noting that the changes in WPD found in this study imply that offshore wind farms have to be efficient both at the current, and at significantly lower than current wind speed levels.

Financial decisions have to take the anticipated decline in energy output (where this happens to be the case) into consideration, and this decline in WPD increases the risk rating for sites which currently are evaluated as having marginal potential for development.

## Supporting Information

S1 File WPD data file. (NC)
